# Recent advancements and limitations of intestinal organoids for clinical applications

**DOI:** 10.1088/2516-1091/ae8107

**Published:** 2026-07-13

**Authors:** Md Zahirul Islam Khan, Somrita Roy, Soumya Nair, Asma Pinkey, Taslim Al-Hilal, Helen Ka-Wai Law, Sourav Roy

**Affiliations:** 1 Department of Biological Sciences, University of Texas at El Paso, El Paso, TX 79968, United States of America; 2 The Border Biomedical Research Center, University of Texas at El Paso, El Paso, TX 79968, United States of America; 3 Department of Environmental Science and Engineering, University of Texas at El Paso, El Paso, TX 79968, United States of America; 4 Departments of Molecular Pharmaceutics and Biomedical Engineering, University of Utah, Salt Lake City, UT 84112, United States of America; 5 Department of Health Technology and Informatics, The Hong Kong Polytechnic University, Hung Hom, Kowloon, Hong Kong Special Administrative Region of China, People’s Republic of China

**Keywords:** organoid, colorectal cancer, 3D culture, application, limitation, drug development

## Abstract

Colorectal cancer (CRC) is one of the leading causes of cancer-related deaths worldwide, and there has been a concerning rise in its incidence among younger populations. Although there have been significant advances in molecular characterization, translating research findings into effective therapeutic strategies for CRC management remains suboptimal. This challenge is largely attributed to the limitations of conventional preclinical models, such as two-dimensional or patient-derived tumor xenograft (PDX), which fail to recapitulate the complexity and heterogeneity of CRC tissues. Intestinal organoids, derived from adult or pluripotent stem cells, have emerged as transformative tools to address the current limitations by mimicking the structural, genetic, and functional characteristics of native intestinal tissue in a three-dimensional culture environment. These organoids preserve patient-specific genomic features, allowing long-term growth, and serve as a more physiologically relevant model for studying CRC initiation, progression and drug resistance mechanisms. Furthermore, the integration of organoids with CRISPR/Cas9 genome editing, high-throughput drug screening, and multi-omics technologies has greatly enhanced their utility in personalized medicine and drug discovery. However, several unsolved challenges remain with the organoid model, such as culturing variability, a lack of protocol standardization, and an incomplete representation of the tumor microenvironment, particularly immune and stromal cells. This review offers a critical and comprehensive overview of intestinal organoid technologies in CRC research, identifies major knowledge gaps, and highlights emerging strategies to enhance their clinical application. The study aims to provide future directions that could significantly enhance precision oncology and ultimately improve therapeutic outcomes for CRC patients.

## 1. Background

Colorectal cancer (CRC) develops from the abnormal growth of polyps in the colon or rectum, which can eventually become cancerous. Globally, CRC is the third most commonly diagnosed cancer, accounting for approximately 10% of all cancer cases. It is also the second leading cause of cancer-related deaths [1]. Recent epidemiological trends reveal a notable decline in CRC incidence among older populations, likely due to advancements in screening and preventive measures. However, there is a concerning shift occurring, which is the dramatic rise in CRC incidence among younger populations. Projections indicate that by 2030, incidence rates in the United States could surge by up to 90% and 124% among individuals aged 20–29 and 30–34 years, respectively [2]. These alarming trends underscore the urgent need for targeted research, revised screening guidelines, and a better understanding of CRC pathogenesis across different age groups.

The heterogeneity of cancer remains the primary obstacle to the development of effective therapeutics across all cancer types [3, 4]. CRC is a highly heterogeneous malignant neoplasm characterized by the abnormal proliferation of cells originating in the intestinal epithelium. These cells undergo excessive proliferation, transitioning from benign adenomas to malignant carcinomas, and may eventually metastasize to distant organs through the bloodstream [5]. This progression results from the cumulative effects of genetic and epigenetic alterations, exposure to carcinogens, and dysregulation of critical signaling pathways, including autophagy, apoptosis, epithelial-to-mesenchymal transition, p53, WNT/*β*-catenin, TGF-*β*-Smads, JAK, AKT, AMPK, MAPK, and Notch [6–8]. The intricate interplay of these molecular mechanisms underscores the complexity of CRC and highlights the challenges in developing universally effective therapies.

Despite significant advances in understanding CRC at the molecular level over the past decade, therapeutic outcomes have not improved proportionally. Although numerous drugs have shown potential anticancer activity *in vitro*, they have failed to demonstrate efficacy in clinical trials [9], emphasizing the gap between preclinical laboratory setups and clinical practice. Traditional laboratory-based anticancer drug development heavily relies on either conventional two-dimensional (2D) cell line models or patient-derived tumor xenograft (PDX) models. Nevertheless, these two models have intrinsic limitations that drastically reduce their effectiveness [10, 11]. For example, extended passaging of traditional 2D cell lines fails to preserve genetic information and heterogeneity in cancer. Whereas PDX studies are unique to mice with different genetic information and a tumor microenvironment (TME) different from that of human tumor patients. The PDX models have other limitations, including poor success rates, low cost-effectiveness, and lengthy transition processes [10, 11]. Therefore, for developing and improving the clinical outcomes of CRC, it is urgent to introduce a new model that can accurately reflect the patient’s genetic information along with superior specificity.

Organoids are self-renewing and self-organizing multicellular masses grown in three-dimensions (3D) in an *in vitro* culture system from stem cells (SCs), including embryonic and pluripotent SCs (PSCs), and can closely mimic the structural and functional characteristics of original the tissues [12–14]. In the specific context of CRC, organoids can also be derived from tumor SCs (TSCs), commonly referred to as cancer SCs (CSCs). CSCs are a hierarchically distinct and functionally privileged subpopulation within the tumor mass that possess self-renewal capacity, multilineage differentiation potential, and the ability to initiate and perpetuate tumor growth, drive metastatic dissemination, and confer resistance to conventional chemotherapy and radiotherapy [15, 16]. In CRC, CSCs are prospectively identified and isolated using a combination of cell-surface markers, most notably CD44, CD133 (Prominin-1), EpCAM, and CD24; these markers enable the enrichment of tumor-initiating populations that can reliably establish organoid cultures *ex vivo* [15–17]. Landmark studies by O’Brien *et al* and Ricci–Vitiani *et al* independently demonstrated that CD133+ cells isolated from primary human CRC specimens could initiate tumor xenografts in immunodeficient mice, thereby formally establishing the CSC concept in CRC [15, 16]. Ricci–Vitiani *et al* further characterized human CRC SCs phenotypically and functionally, demonstrating that EpCAMhigh/CD44+ cells possessed superior tumor-initiating capacity [16]. Because CSC-derived CRC organoids recapitulate the cellular hierarchy, clonal dynamics, and genetic heterogeneity of the parent tumor, they are particularly relevant models for studying tumor initiation, clonal evolution, and mechanisms of therapeutic resistance in CRC. Unlike conventional 2D cell models, the organoid model comprises multiple cell types in a 3D culture environment. As a result, it can accurately simulate the structural and functional characteristics of original tissues to regulate the genetic and phenotypic stability over time [18–20]. Importantly, organoids derived directly from patient or mouse samples overcome the genetic modifications associated with prolonged passaging in 2D cultures. Furthermore, unlike PDX models, organoids are grown entirely *in vitro*, enabling higher-throughput and cost-effective applications in drug testing and personalized medicine [13, 20].

In recent years, tumor organoids have become a hotspot in cancer research, serving as promising tools for modeling a range of diseases, including inflammatory, immunological diseases, and neoplastic conditions. In CRC research, organoids have been instrumental in understanding the molecular mechanisms of tumor initiation, progression, and therapeutic resistance. Additionally, they have facilitated the development of personalized treatment strategies [14, 20–22]. Despite their potential, there are several challenges in applying organoids to CRC carcinogenesis research. These challenges include poor reproducibility, inconsistent culture methodologies, and a lack of standardized protocols. Furthermore, the absence of fibroblasts and immune cells in organoid cultures limits their ability to fully recapitulate the TME *in vitro*. This review offers a comprehensive analysis of the application of human intestinal organoids in CRC research, focusing on culture systems, technical challenges, limitations, and the TME. By addressing these issues, we aim to promote the broader adoption of organoid models and accelerate the translation of findings into clinical practice, ultimately advancing precision medicine for CRC patients.

## 2. History of intestinal organoids

The mammalian epithelium is a highly specialized structure composed of resorptive and secretory cells that protect the columnar epithelium and enable nutrient reabsorption to maintain localized homeostasis. This epithelium also has the potential for self-renewal with an average turnover time of 3–5 d [23]. The villi-crypt architecture serves as the foundation of the intestinal epithelium, regulated by a group of SCs located at the base of the crypts. These SCs undergo differentiation to form mature functional intestinal cells, including absorptive enterocytes, goblet cells, enteroendocrine cells, and Paneth cells [22, 23]. However, the lack of definitive markers and precise localization of these SCs hindered their study for decades. This changed with the groundbreaking discovery of leucine-rich repeat-containing G-protein coupled receptor 5 (Lgr5), also known as G-protein coupled receptor 49 (Gpr49), by Clevers and his colleagues [24].

Lgr5 is a transmembrane G-protein-coupled receptor that enhances Wnt/*β*-catenin signaling by binding to its ligands and R-spondin family glycoproteins [25–27]. This discovery enabled the identification and isolation of Lgr5-positive SCs at the base of intestinal crypts, facilitating their subsequent 3D culture *in vitro* systems. Sato and colleagues pioneered the development of the first 3D intestinal organoid model using isolated Lgr5-positive SCs from mouse intestinal crypts [28]. This was followed by some other groups that have successfully generated organoids and an enhanced culture system using both normal human colon cells and human colon cancer epithelial cells [29–34]. Notably, Lgr5 expression is present not only in the intestine but also in various other tissues, including the liver, tongue, prostate, pancreas, and stomach, thereby enabling the establishment of organoid cultures from diverse tissue types [35].

Based on the regenerative characteristics of human PSCs, organoids can also be derived from PSCs. Following the landmark mouse-derived organoid model, Spence and colleagues developed intestinal organoids from PSCs by mimicking embryonic intestinal development [36]. The process begins with the differentiation of PSC into endoderm, using a series of growth factors and nutrient supplements to mimic embryonic intestinal development. Over time, the cells organize into polarized, columnar structures resembling villus-like projections and crypt-like zones expressing SC markers. This leads to the formation of a functional intestinal organoid. While several studies have since replicated and refined this approach [37–40], challenges remain regarding reproducibility, efficiency, and protocol standardization. Daoud and Múnera have recently provided a comprehensive protocol for generating intestinal organoids from PSCs, addressing common technical challenges, comparing methodologies, and highlighting the advantages and limitations of each [41]. Chen’s group derived colonic organoids from induced PSCs (iPSCs) for modeling CRC and drug screening [42]. They isolated iPSCs from patients with familial adenomatous polyposis, promoted WNT-signaling, and enhanced epithelial cell differentiation to derive successful organoids. The epithelial cells regulate the colonic profiling of the organoid, which is then used in drug screening. These organoids closely recapitulated patient-specific colonic features and were subsequently utilized for high-throughput drug screening. This demonstrates the potential of iPSC-based systems in personalized oncology.

## 3. Intestinal organoid and culture

The Intestinal SCs (ISCs) located at the bottom of crypts facilitate rapid differentiation into different cell types within numerous tissues. This process is driven by the use of reproducible culture conditions that mimic the *in vivo* microenvironment, enabling the maintenance and expansion of ISCs *in vitro*. The stemness of these cells is highly dependent on the activation of the WNT/*β*-catenin signaling pathway, alongside the suppression of bone morphogenetic protein (BMP) signaling through secreted antagonists such as noggin, chordin, gremlin, crossveinless, and follistatin [43, 44]. The crucial WNT and Notch signaling in the small intestine is provided by Paneth cells intercalated between ISCs. These signals are sometimes provided by the stroma and crypt secretory cells in the colon which lack Paneth cells [45–49].

Building on the foundational work of Sato and colleagues, who first developed organoids from mouse small intestine, subsequent studies demonstrated that Wnt3A-conditioned media is not strictly required for the long-term culture of CRC organoids. However, the presence of Wnt3A, p38 MAPK inhibitors, and controlled oxygen concentrations significantly enhances CRC organoid proliferation [28, 50]. For normal colonic organoids derived from mice, advanced DMEM/F12 supplemented with Glutamax and HEPES serves as the base medium enriched with WNT signaling activators such as R-spondin and Wnt3A glycoproteins [51]. This medium also includes epidermal growth factor (EGF) to regulate cell proliferation, rho-associated protein kinase (ROCK) inhibitors to prevent anoikis, and Noggin to suppress BMP-induced differentiation [52, 53].

To maintain organoid growth, B-27, N_2_, N-acetylcysteine, and Nicotinamide are commonly used, and broad-spectrum antibiotics are often added to prevent any bacterial contamination. The common media compositions for successful organoid growth from mouse and human samples are listed in table 1. As the organoids grow in polarized epithelial structures in an adenoma-cancer sequence, the epigenetic modification is negligible and the dependency on niche factors may be compromised to a certain level [30, 50, 54]. Up to the present, numerous research studies have successfully developed CRC organoids that completely replicate the genetic features of the original tumors *in vivo* [50, 55–58]. The application of multi-omics approaches, including proteomics and next-generation sequencing, demonstrated that organoids from primary tumor tissues and tissue-derived organoids can mimic the genetic profile of each other. Numerous relevant studies conducted by different research groups help us to better understand cancer and its microenvironment, thereby providing deeper insights for personalized treatment [56, 59–61].

**Table 1.** Commonly used reagents for intestinal organoid development, culture and maintenance.

Author (year)	Reagents	Human	Animal	References
Sato *et al* (2009)	Advanced DMEM/F12, EGF, R-spondin 1, Noggin, and Y-27632	*X*	Mouse tissue	[28]
Sato *et al* (2011)	Advanced DMEM/F12, HEPES, Glutamax, B-27 supplement, N_2_ Supplement, N-acetyl-L-cysteine, WNT3A, R-spondin 1, PGE2, EGF, FGF-10, Noggin, A83-01, Antibiotics, and Y-27632.	Small intestine, colon and CRC	Small intestine, colon and tumor	[30]
Fuji *et al* (2016)	Advanced DMEM/F12, HEPES, Glutamax, B-27 supplement, Gastrin I, N-acetyl-L-cysteine, EGF, Noggin, R-spondin 1, Wnt-3 A, A83-01, Antibiotics, and SB202190.	Colon tissues and CRC	*X*	[50]
Buzzelli *et al* (2018)	DMEM/F12, Glutamax, StemPro, R-spondin 1, Noggin, EGF, IGF-1, FGF-10, FGF*β*, ETC, and Y-27632.	Human CRC with liver metastasis	*X*	[62]
Toden *et al* (2018)	Advanced DMEM/F12, L-glutamine, FBS, L-WRN media, SB431542, Y-27632, Primocin and Antibiotics	CRC	CRC	[63]
Mukohyama *et al* (2019)	Advanced DMEM/F12, Glutamax, HEPES, FBS, ITES supplement, EGF, R-spondin 1, Noggin, Sodium pyruvate, Amphotericin-B, Y-27632, and Antibiotics	CRC, cell lines	Patient derived xenografts	[64]
Jelinsky *et al* (2022)	Advanced DMEM/F12, Glutamax, HEPES, B-27 supplement, EGF, Gastrin, Noggin, Nicotinamide, R-spondin 3, iWP2, DAPT, Wnt3a conditioned media, SB202190, PD0325901, Primocin, N-acetyl-L-cysteine, A83-01, and Antibiotics	Inflammatory bowel diseases derived colon	*X*	[65]

**Figure 1.**
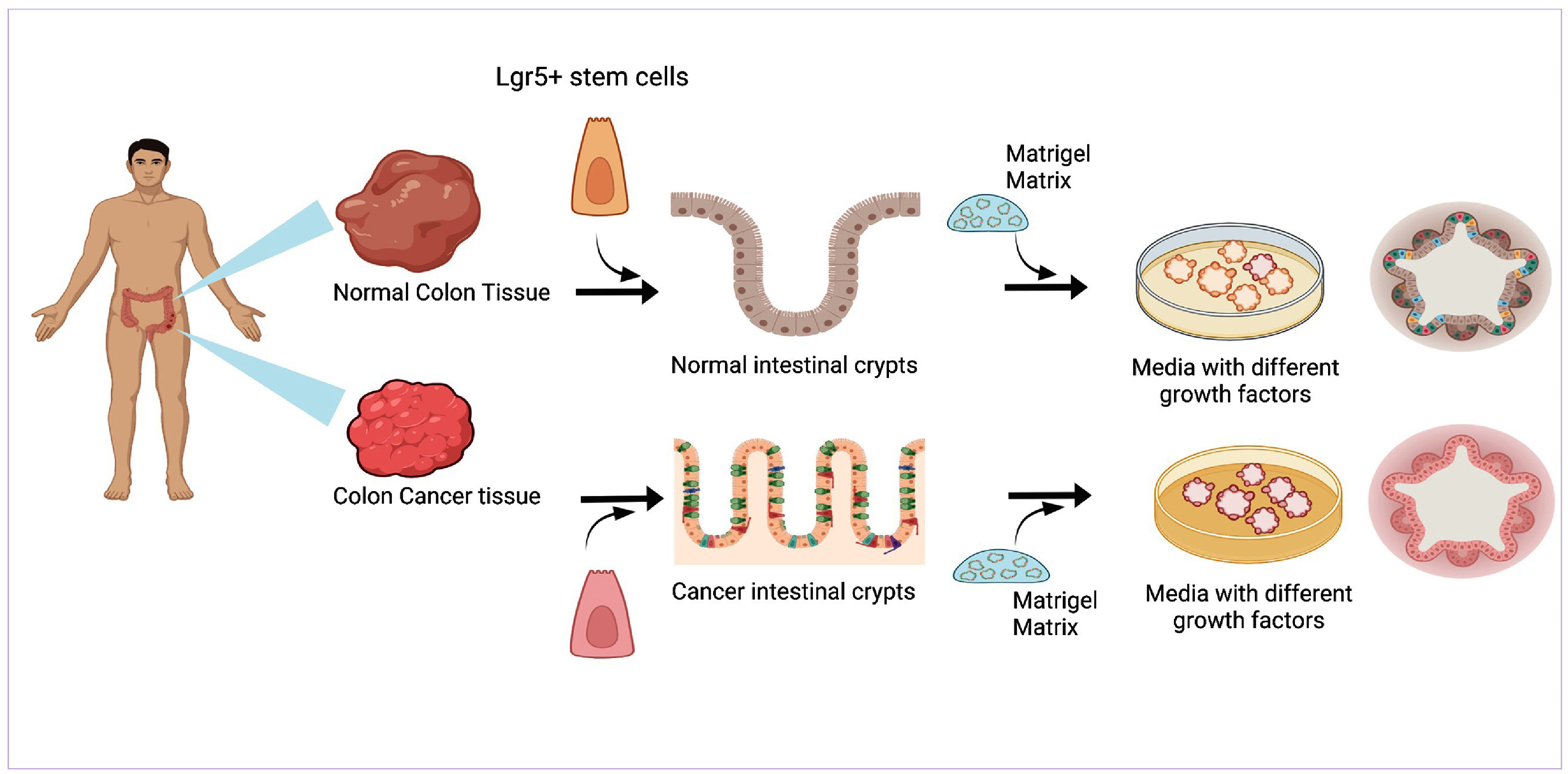
Organoid construction from normal and cancerous intestinal tissues. Derivation of normal and CRC organoids from patient biopsy or surgical specimens through the isolation of LGR5^+^ ISCs or TSCs, followed by embedding in basement membrane matrix (Matrigel) and three-dimensional expansion under defined culture conditions supplemented with niche factors.

CRC organoids are typically established from surgical resections, endoscopic biopsies, or tissue-derived SCs, where modifications in the normal human colon tissue culture media and its supplements are commonly utilized to optimize growth and differentiation. A schematic representation of organoid development from normal and cancerous tissues is presented in figure 1.

Despite significant progress, there is currently no universally accepted protocol for growing CRC organoids. Existing methodologies primarily rely on two systems: 1) growth on flat matrix gels, which facilitate imaging and tracking but suffer from low throughput and reproducibility; 2) polystyrene-coated polydimethylsiloxane microporous matrices, which support high-throughput clonal expansion but may impair organoid development [22]. Innovations such as type I collagen gels have been introduced to overcome limitations in high-throughput drug screening, further expanding the utility of organoid models in preclinical research [22]. Genomic analyses reveal that over 94% of CRCs harbor mutually exclusive mutations in key regulators of the WNT/*β*-catenin pathway, including APC, CTNNB1, or AXIN. This leads to continuous activation of this constitutive pathway in tumor organoids derived from CRC patients (PDTOs). Therefore, Wnt3A-conditioned media is not mandatory for their culture [66–68]. Sato and colleagues further refined organoid culture media by incorporating Wnt, SB202190 (a p38 MAPK inhibitor), growth factors, and optimizing oxygen levels, achieving nearly 100% organoid development efficiency in compared to the conventional 70% success [50, 69]. Their work also demonstrated the successful generation of organoids from rare colon neuroendocrine tumors, highlighting the versatility of this technology. The overall findings highlight that progressive acquisition of genetic and epigenetic alterations during tumor evolution further reduces the dependence on extrinsic niche factors, thereby enabling sustained organoid growth under less complex culture conditions [50, 54, 70, 71].

## 4. Applications of intestinal organoid

**Figure 2.**
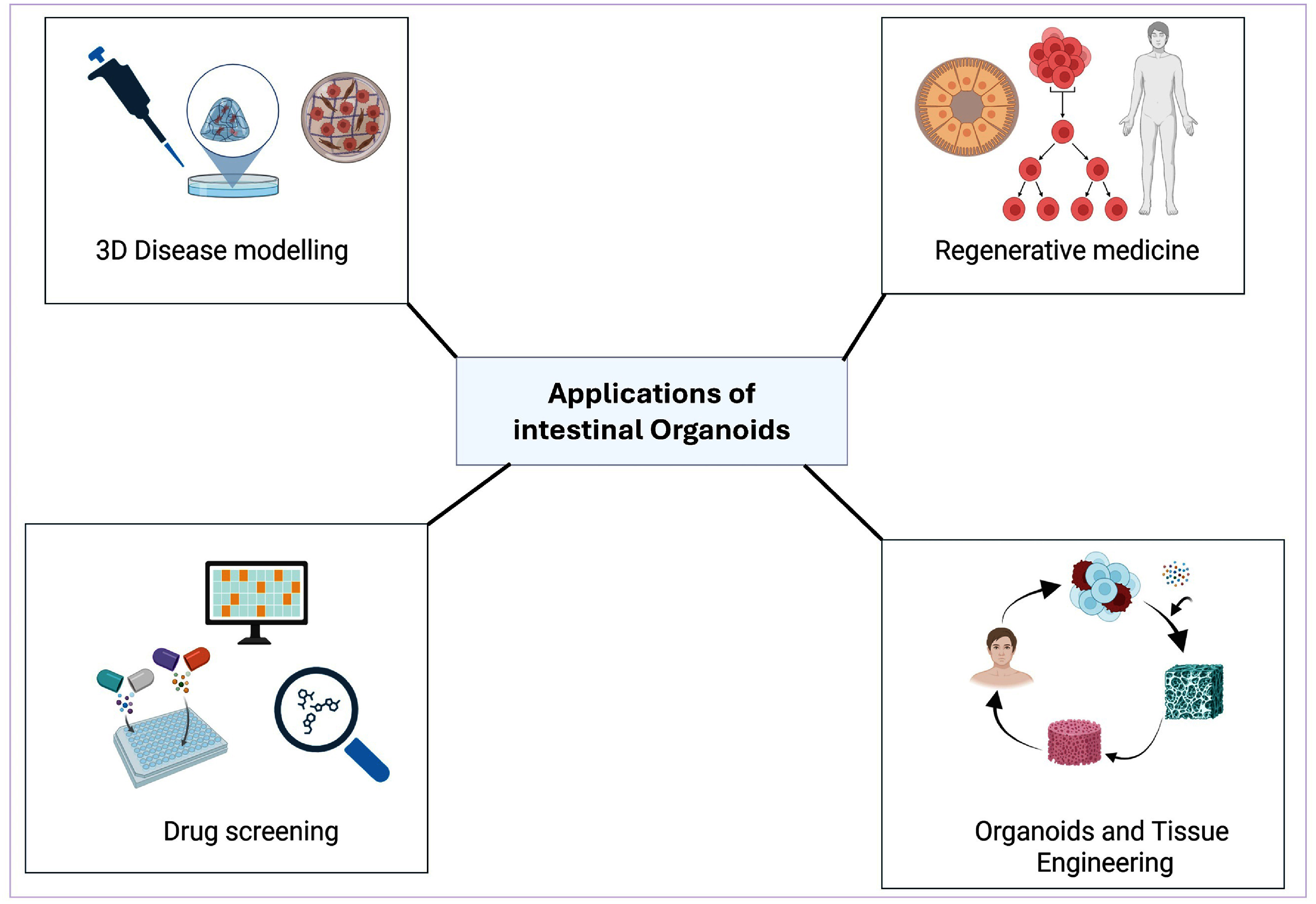
Clinical applications of intestinal organoids. Overview of the major applications of intestinal organoids, including disease modeling, drug screening and personalized medicine, regenerative medicine, and organoid engineering for translational and therapeutic research.

Intestinal organoids have emerged as transformative tools in life science research, providing unique opportunities to study human biology, model diseases, and advance therapeutic development. These 3D *in vitro* systems replicate the structural and functional complexity of native tissues, addressing many limitations of traditional models such as 2D cell cultures and PDX. Below, we explore the key applications of intestinal organoids in disease modeling, regenerative medicine, drug screening, and organoid engineering, highlighting their potential to revolutionize biomedical research (figure 2 and table 2).

**Table 2.** Application of intestinal organoids.

Species	Organ/ type	Cell sources	Applications	References
Human, rat, and dog	Intestine	LGR5+ and iPSCs	Disease modeling and genetical characteristics evaluation	[72–74]
Human and mouse	Human intestinal mucosal biopsies and mouse intestine	LGR5+ and SCs	Evaluation of epigenetic dysregulation associated with severe Crohn’s Diseases	[75]
Human	Human intestine	LGR5+ cells	Transcriptomic evaluation and biomarker establishment for of inflammatory bowel diseases	[76]
Mouse	Mouse colon	LGR5+ cells	Tissue engineering	[77]
Human	Biopsies from CRC patients	Not mentioned	Anticancer drugs screening	[78]
Human and mouse	Human and mouse intestine	Not mentioned	Drug library (335 anticancer drugs) screening to get the potential candidates	[79]
Human	Small intestine from gastric bypass surgery	Colonic SCs	The cutting-edge micro levels of bioengineering on lab-on-chip model study	[80, 81]
Human and mouse	Intestine	LGR5+ cells	CRISPR-Cas9-based gene engineering	[54, 82]
Human organoid and cells	CRC organoid and cells	Not mentioned	Toxicological study	[83, 84]

### 4.1. Modeling of diseases

Organoids serve as highly accurate *in vitro* replicas of native tissues, making them ideal for disease modeling. While animal models provide insights into systemic responses, differences between these models and human physiology often contribute to clinical trial failures. Organoids address several limitations of traditional cell line studies and have become indispensable tools for studying complex diseases, especially cancer.

Derived from tissue-specific multipotent SCs and their differentiated progeny, organoids exhibit self-organization and can be cultured indefinitely under appropriate conditions [20, 33, 85, 86]. The human intestinal organoids were the first to be successfully developed *in vitro* [29–34]. Organoid protocols have since been refined to generate models from diverse tissues, including the pancreas, liver, kidney, stomach, and lungs [87–91]. These self-organizing structures exhibit long-term expansion potential under defined culture conditions; however, it is critical to emphasize that the proliferative capacity of organoids *in vitro* is inherently limited and that these are fundamentally distinct from and should not be considered equivalent to immortalized cell lines [9, 11, 13]. Unlike immortalized cell lines, which acquire the ability to proliferate indefinitely as a result of genetic alterations that abrogate normal growth restraints (e.g. mutations in TP53, activation of telomerase, and dysregulation of cell cycle checkpoints), organoids retain near-normal chromosomal stability, have finite passaging capacity without phenotypic or genetic drift, and require periodic re-establishment from cryopreserved stocks or fresh patient tissue [13, 86]. This biological distinction is of critical importance for experimental planning, as it imposes practical constraints on the number of achievable passages and necessitates more stringent quality-control measures. Nevertheless, precisely because organoids maintain greater physiological fidelity than immortalized cell lines, they remain superior and more clinically relevant models for studying intestinal function, disease phenotypes, and drug responses. Human intestinal organoids, in particular, reproduce essential features such as crypt-villus architecture and maintain functional subsets of intestinal epithelial cells, including enterocytes, goblet cells, Paneth cells, and ISCs [34, 92]. Recent research has demonstrated the exceptional adaptability of organoids for live-cell imaging, metabolic activity assessments, genome-wide gene expression analyses, protein profiling, and gene-editing technologies [86, 93–95]. Their compatibility with high-throughput assays while preserving the functional properties of 3D tissues makes them invaluable for studying intestinal function, modeling disease phenotypes, and conducting drug discovery screens [61, 96, 97].

A major translational advancement in CRC research has been the establishment of patient-derived organoid (PDO) biobanks, renewable repositories of tumor-derived organoids that collectively capture the genetic and phenotypic diversity of CRC across patient populations. Van de Wetering and colleagues pioneered the first living CRC organoid biobank comprising organoids from 20 CRC patients, demonstrating that these organoids faithfully recapitulate the somatic mutational landscape of their source tumors, including canonical driver mutations in APC, TP53, KRAS, and SMAD4 [34]. This biobank-based approach has since been substantially scaled up; Luo *et al* established a large-scale patient-derived high-risk colorectal adenoma organoid biobank enabling high-throughput and high-content drug screening, identifying candidate therapeutic agents with subtype-specific activity [98]. More recently, Farin and colleagues developed a CRC organoid–stroma biobank that co-cultured patient-derived tumor organoids with cancer-associated fibroblasts (CAFs), enabling subtype-specific assessment of individualized therapy responses and revealing the influence of stromal composition on drug sensitivity [99]. Patient-derived organoid biobanks have also demonstrated predictive power in clinical settings; Vlachogiannis *et al* showed that organoids derived from patients with metastatic gastrointestinal cancers accurately predicted individual responses to targeted therapies in clinical trials [100]. Collectively, CRC organoid biobanks represent a powerful infrastructure for biomarker discovery, mechanistic studies of inter-patient heterogeneity, and functional precision oncology at scale.

### 4.2. Regenerative medicine

Organoids exhibit a remarkable ability to recapitulate the tissue-specific architecture and cellular heterogeneity of their tissue of origin, establishing them as a transformative platform in regenerative medicine and disease modeling. Organoids can be derived from minimal patient tissue through minimally invasive procedures and expanded robustly *in vitro*, making them highly suitable for autologous transplantation and personalized therapeutic strategies [101, 102]. Organoids generated from tissue-specific adult SCs (ASCs) can differentiate into relevant cell types, directly contributing to tissue regeneration upon transplantation, thereby, enhancing the efficacy of regenerative interventions [102, 103]. ASC-derived organoids present a lower risk of tumorigenicity compared to PSCs-derived models. Additionally, their ability to match the patient’s own tissue reduces the likelihood of immune rejection. Direct transplantation of organoids to sites of injury is often achievable via minimally invasive, image-guided techniques that reduce the risk of ectopic engraftment and organ-to-organ spread in clinical uses [101, 104].

Preclinical studies have demonstrated the feasibility and therapeutic potential of organoid transplantation. For example, orthotopic transplantation of epithelial organoids into injured murine colon have facilitated donor-derived epithelial regeneration. Additionally, human intestinal organoids have restored epithelial function and lineage integrity in immunodeficient mice with mucosal injury [105]. Colon organoids have proven effective in reversing DSS-induced colitis, underscoring the plasticity and transplantability of the colonic epithelium [57]. Recent studies suggest that transplanted organoids alleviate intestinal ischemia-reperfusion injury by promoting ISC renewal and modulating the immune microenvironment, with L-malic acid secretion mediating M2 macrophage polarization in a SOCS2-dependent manner [106].

Recent advancements in bioengineering have facilitated the transplantation of larger tissue blocks through vascularization [107]. Combining organoids with 3D bioprinting technologies, researchers have created centimeter-scale tubular intestinal tissues with arterial networks, connective tissue, and glandular-villus formations [108]. Researchers have suggested using organoids and 3D bioprinting technologies to create a tiny gut. Sugimoto and colleagues generated functional colons resembling the small intestine using ileum-derived organoids, offering a viable approach for treating short bowel syndrome [109]. Together, these findings underscore the clinical significance of organoid transplantation and biofabrication in gastrointestinal regenerative therapies.

### 4.3. Drug screening

Organoid cultures have rapidly emerged as a transformative platform for preclinical drug screening due to their unique ability to absorb and metabolize drugs, nutrients, and water [72]. Along with the traditional cell lines used in drug screening, animal models, including rodents, dogs, and monkeys, have been considered; however, significant anatomical, physiological, and biochemical differences, such as variation in pH [34], digestive fluid composition, and microbiome content, render these models suboptimal for accurately predicting human responses [110, 111]. Since 2015, several research groups, such as Clever’s group (41 cases) [34], Greten’s group (30 cases) [99], and Liu’s group (33 cases) [98], have successfully developed PDOs, creating models that closely mirror tumor biology. These PDOs provide a valuable platform for high-throughput drug screening, enabling researchers to evaluate therapeutic responses more accurately and potentially personalize treatment strategies. Among those, studies by Clevers and colleagues’ are considered most groundbreaking, where they optimized intestinal organoids for high-throughput drug screening, and evaluated them in 384-well plates against 83 diverse drugs [34]. This panel consisted of 10 chemotherapeutics, 25 drugs already in clinical use, 29 investigational drugs, and 29 targeted therapies. Their findings demonstrated a significant correlation between the genotypic profiles of PDOs and their drug sensitivities. TP53-mutated tumor organoids showed resistance to MDMI inhibitors, and KRAS-mutated organoids displayed resistance to ERBB inhibitors [34]. Similarly, Cartry and colleagues screened an array of 25 approved anticancer drugs in 25 PDOs. Their PDO-based drugs screen demonstrated potential clinical relevance, achieving 75% sensitivity and specificity in predicting therapeutic responses, suggesting the effectiveness of PDOs as a predictive model in oncology [78]. Another study developed a robust organoid-based drug-screening platform using eight CRC organoids. A total of 335 approved small-molecule drugs and computational analysis-based target drugs were tested. Finally, 34 drugs show notable effectiveness against CRC models using this combination strategy, highlighting the promise of organoid-based techniques in drug screening, development, and precision oncology [79]. A pivotal study by the University of Basel and Novartis Institutes for Biomedical Research harnessed intestinal organoids for high-throughput drug screening, revealing intricate phenotypic profiles and shedding light on the effects of RXR receptor inhibitors on organoid regulation [112]. The researchers analyzed functional gene interactions that are essential for organoid development and regenerative potential. They categorized the compound-treated organoids into 15-element fingerprint clusters which could be targeted for further drug screening. This study demonstrated that a multivariate phenotypic screening approach can unravel the complex mechanisms underlying intestinal regeneration, providing new insights into regenerative medicine [112]. Overall, intestinal organoids serve as effective tumor models, enabling rapid, animal-free drug screening to assess antitumor efficacy and toxicity, accelerating safe drug development.

### 4.4. Organoid engineering

The intestinal mucosa comprises diverse cell types, that are vital to its biological and physiological properties; however, replicating this complexity remains a challenge *in vitro*. The discovery by Barker’s lab of Lgr5+ SCs as drivers of epithelial regeneration within the crypt-villus structure was a guideline for further development [24]. This discovery enabled the generation of patient-derived ASC-mediated small intestinal organoids that can replicate the crypt-villus architecture *in vitro* [28, 30, 45]. Similarly, the PSC-derived organoids have shown successful *in vivo* engraftment. This approach serves as an alternative to limited access to gut tissue, fostering mesenchymal cells that support the niche environment [36, 113, 114]. Taking high incidence and mortality of CRC into consideration, extensive research has focused on modeling oncogenic transformation, where CRISPR/Cas9 eased the process by enabling mutation introduction to mock up the cancer progression [54, 70, 115]. A recent study demonstrated that through tissue engineering and self-organization, ISCs can generate tube-shaped epithelia with a luminal structure and spatially distributed crypt- and villus-like domains [116]. Brassard and colleagues implemented a combined approach of bio-fabrication with organoid technology, specifically 3D bioprinting, which enables precise cell deposition into an extracellular matrix (ECM) that promotes intrinsic self-organization and advanced organoid structure and function [108]. The application of an organ-on-a-chip system enabled monitoring various intestinal cell types with their structural and functional complexities, facilitated studies on intra- and inter-organ cell-to-cell interactions [117, 118]. Embedded sensor systems enable the precise measurement of physiological characteristics, facilitate studies of host microbiota, assess oxygen gradients, and monitor peristaltic movements of cells, ultimately tracking cellular behavior and functions [119–121]. Beyond complex studies, the simple organoid system can effectively monitor specific cellular characteristics. For example, Hackam’s lab demonstrated that intestinal organoid proliferation and Paneth cell differentiation are potentially improved when organoids are co-cultured with commensal bacteria [122]. Similarly, another study introduced the pathogenic parasite Cryptosporidium into organoid culture to monitor pathogen-epithelium interactions, infection mechanisms, and epithelial responses to the pathogen [123]. Overall, organ-on-a-chip systems offer versatile platforms for exploring complex cell-cell and organ interactions, physiological conditions, and microbiota-host dynamics, advancing insights into intestinal biology and diseases.

### 4.5. Gene editing and CRISPR/Cas9 applications

Gene editing technologies, in particular the CRISPR/Cas9 system have fundamentally transformed the utility of intestinal organoids by enabling precise, programmable modifications to the organoid genome in a physiologically relevant epithelial context. Intestinal organoids are uniquely amenable to CRISPR-based engineering owing to their clonal expansion from single cells, high transfection efficiency via electroporation or lipofection, and their ability to recapitulate *in vivo* tissue architecture following genetic manipulation [33, 95]. In CRC research, the organoid–CRISPR paradigm was pioneered by Matano and colleagues, who used sequential CRISPR/Cas9-mediated engineering of normal human intestinal organoids to introduce five key CRC driver mutations, inactivation of APC, SMAD4, TP53, and FBXW7, and activation of KRAS thereby generating a de novo human CRC organoid model that faithfully recapitulated the adenoma-to-carcinoma sequence *in vitro* [54]. Simultaneously, Drost and colleagues modeled the sequential accumulation of cancer driver mutations in cultured human ISCs using CRISPR/Cas9, demonstrating the utility of organoids for studying clonal evolution and the mutational signatures underlying CRC initiation [70]. Beyond disease modeling, CRISPR/Cas9 has been applied in organoids as a proof-of-concept platform for therapeutic gene correction. Schwank and colleagues demonstrated functional repair of the CFTR mutation in patient-derived intestinal organoids from cystic fibrosis patients using CRISPR/Cas9-mediated homologous recombination, establishing the feasibility of organoid-based gene therapy modeling [124]. The integration of more recent genome editing modalities including base editing, prime editing, and homology-independent targeted integration has further expanded the editing toolkit available for organoid-based applications. Geurts and Clevers systematically reviewed these CRISPR engineering strategies in organoids, highlighting their growing applications in disease modeling, functional gene discovery, and therapeutic gene repair across multiple organ systems [115]. An emerging and particularly powerful application is the use of genome-scale CRISPR screening in CRC organoids for functional interrogation of genetic dependencies. Drost *et al* used CRISPR-modified human ISC organoids to study the origin of mutational signatures in cancer, revealing distinct endogenous mutagenic processes relevant to CRC etiology and demonstrating the power of organoid-based systems for modeling complex cancer genetics in a physiologically authentic context [125]. Collectively, the breadth of gene editing applications ranging from de novo disease modeling and mutation discovery to therapeutic correction and functional genomic screening positions CRISPR/Cas9-engineered intestinal organoids as indispensable platforms for advancing precision oncology in CRC.

### 4.6. Co-culture systems, vascularization, and multi-organ platforms

A rapidly expanding frontier in organoid technology involves integrating immune cells, vascular networks, and multi-organ platforms to generate more physiologically complete and clinically representative models. To address the absence of immune components in conventional organoids, researchers have developed immune–organoid co-culture systems that enable the study of host–pathogen interactions, inflammatory responses, and tumor–immune dynamics *in vitro*. Bouffi and colleagues demonstrated that human intestinal organoids transplanted into humanized mice develop a tissue-resident immune compartment that closely recapitulates the *in vivo* immune cell diversity, organization, and function [55]. More recently, Recaldin *et al* engineered human organoids with an entirely autologous tissue-resident immune compartment generated *in vitro*, enabling direct investigation of immune–epithelial crosstalk, antigen presentation, and mucosal immunity without the need for animal transplantation [126]. These immune co-culture systems are particularly valuable for modeling inflammatory bowel diseases, infectious enteropathies, and tumor immune evasion in the context of CRC. Vascularization of organoids is another key application area that addresses a fundamental structural deficiency of conventional organoid models. Nwokoye and Abilez systematically reviewed bioengineering strategies for vascularizing organoids, encompassing co-culture with primary endothelial cells, integration within perfusion-based microfluidic systems, and the use of vascularized bioengineered scaffolds [107]. These vascularization approaches not only improve nutrient delivery, oxygen diffusion, and waste removal; overcoming hypoxia-induced necrosis in organoid cores but also enable studies of angiogenesis, vascular co-option, and systemic drug pharmacokinetics that are directly relevant to CRC progression and therapy. Multi-organ interaction platforms offer yet another powerful application for intestinal organoids. Integration of intestinal organoids within organ-on-a-chip systems has facilitated controlled studies of gut–liver axis function, host–microbiome dynamics, and inter-organ drug metabolism [117, 118]. These microfluidic platforms embed organoids in biomimetic flow conditions that impose physiologically relevant shear stress, oxygen gradients, and paracrine signaling. Additionally, assembled technologies involving the physical fusion of organoids from different tissue lineages offer complementary approaches to model regional inter-organ interactions, including CRC hepatic metastasis and systemic inflammatory responses [127]. Together, these co-culture, vascularization, and multi-organ applications position intestinal organoids at the forefront of next-generation physiological modeling, bridging the gap between reductionist *in vitro* systems and complex *in vivo* biology.

## 5. Limitations of organoid study

**Figure 3.**
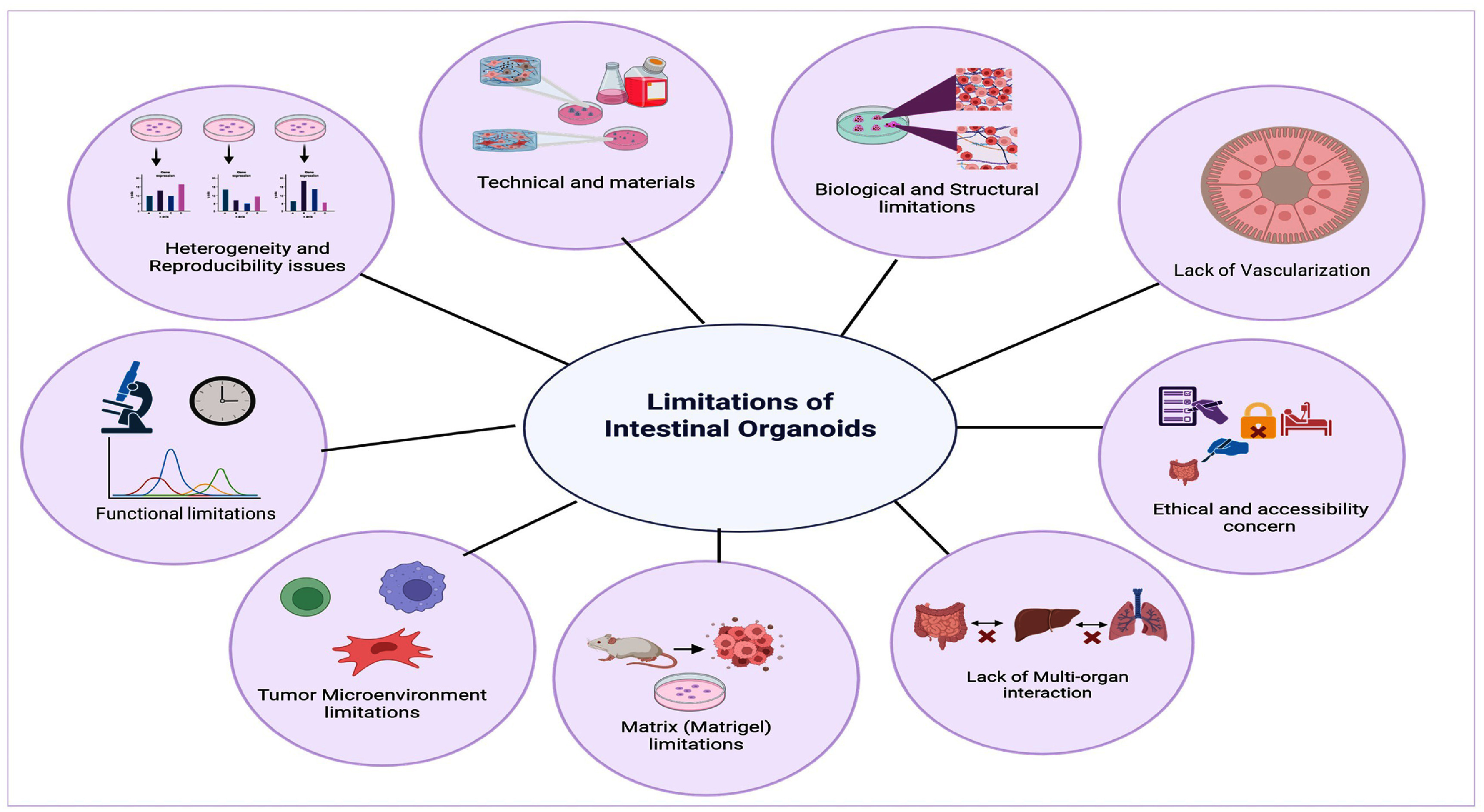
Major limitations of intestinal organoids. Schematic overview of the major limitations associated with intestinal organoid models, including biological and structural challenges such as the absence of vasculature, immune cells, and stromal components; incomplete recapitulation of the tumor microenvironment; technical and material limitations including Matrigel variability and culture heterogeneity; and ethical considerations related to organoid research and clinical translation.

Intestinal organoids are a powerful model for studying cancer biology, disease modeling, and drug testing. However, they have notable limitations (figure 3) in completely mimicking the complex *in vivo* environment of human tissues due to the absence of key components, including blood vessels, immune cells, and nervous system associated with it. The details of the limitations are described below.

### 5.1. Biological and structural challenges

Organoids serve as a relevant model of human organs, but they fail to fully replicate the complexity, maturity, and functionality of real organs. This limitation arises due to inadequate cellular organization, including the absence of mesenchymal compartments, vascularization, and microbiomes [128]. Organoids derived from PSCs often lose their proliferative capacity and fail to mature beyond a certain stage [82]. Similarly, ASC-derived organoids consist primarily of epithelial cells, and lack the diverse cellular components necessary for full organ functionality. For instance, iPSC-derived intestinal organoids predominantly differentiate into small intestine-like structures rather than the colon and cecum, requiring additional components to modulate the colonic signaling pathways, such as the BMP pathway, to generate colonic organoids [129]. In addition to structural limitations, organoids also lack functional immune components and physiological networks, reducing their relevance in research applications. This functional immaturity can lead to inaccurate predictions of drug metabolism, toxicity, and therapeutic responses [130].

**Heterogeneity and reproducibility issues:** Organoids display considerable heterogeneity both within individual cultures and among between cultures. While the organoids may contain all the necessary cell types for an organ, these cells are not always present in consistent proportions across different organoids [131]. Such variability and heterogeneity among the organoids affect experimental reproducibility, making it challenging to derive reliable conclusions, particularly in drug testing. Additionally, established patient-derived organoids may show diverse phenotypes, further complicating the interpretation of experimental results [132].

**Limitations in modeling the TME:** Despite providing a 3D model of tissues, organoids fail to accurately represent the TME. They typically lack components of native tissue’s stromal and immune components, consisting primarily of epithelial tumor cells [133]. Principal components in the TME, such as tumor-associated macrophages (TAMs) and CAFs, are absent, even though these cells are critical for modulating tumor responses to therapy [134]. This absence limits the ability of organoids to replicate *in vivo* microenvironments where immune interactions, nutrient exchange, and support from surrounding tissues influence cellular behavior and treatment responses. To address these concerns, researchers have attempted to supplement organoid cultures with exogenous components, such as peripheral blood mononuclear cells, primary leukocytes, TAMs, and CAFs. The absence of stromal, immune, and vascular components significantly limits organoids’ utility in studying immunotherapy, tissue repair, and disease progression [135]. Stromal cells, a major cellular component, maintain the structural integrity of tissues and regulate various physiological processes, including tumor metabolism, growth, metastasis, immune evasion, and treatment resistance [136]. Without stromal cells in organoid cultures, critical signaling pathways among tumor, immune, and stromal cells in the TME could be disrupted, ultimately affecting disease progression and clinical outcomes in both experimental and clinical settings [136].

**Immune system deficiencies in organoids:** The intestinal mucosa houses the largest immune cell population in the human body, playing a significant role in cellular homeostasis and disease progression [126]. However, the intestinal organoids lack specialized immune compartments, which limit their ability to model immune interactions. As a result, organoids do not fully capture the complex dynamics of immune cells in conditions such as inflammation and cancer. For instance, in cancer research, the lack of immune components in organoids can reduce their applicability for testing immunotherapies or understanding tumor-immune system interactions [137].

**Lack of vascularization:** A functional vascular system is essential for nutrient transport, oxygenation, waste removal, and tissue homeostasis [138]. However, organoids lack blood vessels, leading to hypoxia and nutrient deprivation in their core. This limitation results in necrosis, impaired cellular functionality, and an inability to accurately model disease progression, particularly in cancer research [107]. Without an established vasculature, organoids can only mimic early-stage tumors and not the complexities of advanced cancers [139]. Some research efforts are focused on integrating vasculature using co-culture systems or bioengineered scaffolds, but this area still requires further innovation [127].

**Lack of multi-organ interaction:** Organoids are designed to model single tissue types, limiting their ability to study interactions between different organs. This is particularly problematic for diseases involving multiple organ systems or where inter-organ communication plays a critical role, such as metastatic cancer and systemic inflammatory diseases [137]. While emerging technologies such as assembloids (fused organoids from different lineages) offer a way to model regional interactions, they still fall short of capturing the complexity of *in vivo* multiorgan communication. Additionally, organoids often lack endocrine and nervous system components, making them inadequate for studying systemic disease responses [127]. A potential solution is the integration of organ-on-a-chip technology, which enables controlled interactions between different tissue models within a single system.

### 5.2. Technical and material challenges

**Optimization of culture conditions and growth factors:** The development and functionality of organoids are significantly influenced by culture conditions and the components used for their growth. However, current protocols vary widely and utilize different compositions of growth factors and ECM components, often customized to specific study objectives [137]. This inconsistency in culture conditions inhibits the ability to fully replicate native tissue environments, limiting the reproducibility and physiological relevance of organoid models. The development of organoids relies on a complex interplay of signaling molecules, ECM interactions, and cell-to-cell communication, all of which depend on the culture media. For organoid cultures to maintain diverse tissue cell types and ensure reproducibility, they require an optimal balance of signaling molecules such as WNT and EGF. Excessive concentrations of these signals can disrupt cellular differentiation, leading to the loss of certain differentiated cell populations and subsequently cell homogeneity [140]. The lack of optimized culture conditions contributes to inconsistencies in organoid size, shape, and functionality, ultimately affecting experimental outcomes [141].

**Growth factors in organoid culture:** Organoid culture media often contain unnaturally high concentrations of growth factors to support cell survival and proliferation *in vitro*. However, these elevated levels differ significantly from the balanced and regulated growth factor levels found in the human body. While these formulations promote organoid growth, they can also lead to artificial cellular responses, exaggerated growth rates, and unrepresentative responses to drugs and therapies, which may not translate effectively *in vivo* [86]. This limitation is problematic in cancer research, where accurately modeling tumor progression and drug responses is critical for mimicking physiological conditions. For instance, prostate cancer organoids grown in excessive growth factor conditions may exhibit cellular behaviors that do not accurately reflect *in vivo* tumor biology, thus impacting drug testing and treatment strategies [137]. Consequently, preclinical drug testing conducted under these artificial conditions may yield results that fail to translate effectively into clinical settings. The reliance on growth factor-rich media also complicates the transition of organoid models from research to clinical settings [139]. The *in vivo* physiological conditions must be closely mimicked to ensure the predictive accuracy of these models for drug development and personalized medicine. To address these challenges, ongoing research is focused on optimizing growth factor concentrations, incorporating gradient-based growth factor delivery systems, and developing more physiologically relevant culture media that better mimic native organ environments.

Matrigel, a commonly used ECM scaffold derived from mouse sarcoma cells, is crucial for supporting organoid growth. However, it presents several challenges, including batch-to-batch variability, ethical and immunogenic concerns, and limited scalability. Matrigel’s undefined and complex composition leads to inconsistencies across batches, affecting the reproducibility of organoid studies. This batch-to-batch variability is particularly challenging for high-throughput applications, including drug screening and precision medicine [137]. As an animal-derived product, Matrigel raises ethical concerns and presents risks of immunogenic reactions or pathogen contamination, which pose regulatory challenges for clinical application [139]. The reliance on Matrigel limits the scalability and adoption of organoid technology for clinical and industrial applications, where standardized and reproducible ECMs are necessary. To decrease the dependency on animal products, alternatives to Matrigel can be used, such as synthetic hydrogels, which can mimic human ECM properties and enhance reproducibility and scalability [86].

To overcome the limitations of Matrigel, researchers are actively exploring alternative ECM scaffolds, including natural, hybrid, and synthetic hydrogels. Natural hydrogels, derived from biological sources, provide excellent biocompatibility but often suffer from batch-to-batch variability and rapid degradation, making them less suitable for standardized organoid research [128]. Synthetic hydrogels, such as those based on polyethylene glycol, allow for precise control over physical and chemical properties, making them highly scalable and reproducible. However, synthetic matrices often lack the biochemical cues necessary for organoid differentiation and functional maturation. This limitation can hinder their ability to fully replicate the native tissue environments, thereby affecting the relevance of organoids in clinical research [142]. While synthetic hydrogels are increasingly being engineered to mimic specific tissue ECM properties, challenges remain in achieving the ideal balance between biocompatibility, bioactivity, structural support, and long-term stability seen with natural matrices [143, 144]. Hybrid hydrogels, which combine natural and synthetic components, aim to balance biological compatibility with structural stability. Hydrogels provide increased precision and spatiotemporal release of bioactive elements, as in natural ECMs, and they have consistency and tunable physical properties as in synthetic materials. This combination allows for improved reproducibility while still promoting essential cellular interactions [145]. Ongoing research aims to optimize hydrogel formulations to create more physiologically relevant organoid models that better replicate *in vivo* conditions, ultimately improving their biomedical applications.

### 5.3. Practical and experimental challenges

**Low efficiency and variability in organoid establishment:** The success rate of organoid establishment varies significantly across different tissue types, ranging from 15% to 90%. This variability stems from multiple factors, including the condition and quality of the starting material, culture protocols, and the intrinsic biological differences among tissues [143]. For example, deriving organoids from primary prostate cancer often yields low efficiency, and fibrotic or necrotic tissues further challenge successful organoid formation due to their altered cellular and extracellular properties [137]. These inconsistencies limit the reliability of organoids in applications requiring high reproducibility, such as drug testing and personalized medicine. Additionally, low success rates limit the ability to generate a diverse set of organoid models that accurately represent different disease stages and patient profiles [139]. Efforts to enhance organoid establishment focus on optimizing culture conditions, selecting the most viable tissue sources, and improving cryopreservation techniques to maintain cell viability. Despite these advancements, inherent biological and technical limitations continue to challenge efficiency [86]. However, technologies, including single-cell sequencing and advanced imaging, offer potential strategies to improve success rates by providing deeper insights into factors influencing organoid formation.

**Reproducibility issues in organoid size, shape, and cellular composition:** Reproducibility remains a major challenge in organoid research, as organoids frequently exhibit significant variation in size, shape, and cellular composition. These inconsistencies arise from biological variability in SCs, differences in culture conditions, and the stochastic nature of organoid formation [143]. Such variability impacts reproducibility and complicates the standardization of organoid-based assays, particularly in high-throughput drug screening [137]. Even organoids derived from the same tissue sample can display diverse morphologies, affecting experimental consistency and complicating data interpretation. Additionally, differences in cellular composition influence the ability of organoids to accurately replicate disease states, reducing their reliability as preclinical models [139]. Efforts to improve reproducibility include refining culture protocols, implementing standardized methodologies, and utilizing advanced imaging and analytical tools to monitor consistent organoid formation and characteristics [144]. Furthermore, research has shown that alterations in transcriptional profiles in organoids are often associated with culture conditions, growth media, and donor variability more than with disease status. Addressing the variabilities in organoid development, culture conditions, and morphological variability is crucial for improving the standardization of organoid-based models for translational research and clinical applications.

**Limitation of functional readouts:** The functionality of organoids is typically assessed through specific readouts, such as the metabolic activity of endogenous or exogenous compounds, peptide secretion, and cellular responses to external stimuli. However, these functional assessments often fall short of capturing the full complexity of organ behavior, primarily due to the heterogeneous nature of organoid formation [128]. For instance, a common approach involves the use of optical and fluorescence microscopy to monitor cell behavior and responses; however, these methods are typically limited to capturing single time-point measurements. As a result, experiments often need to be interrupted to obtain data, limiting the ability to monitor real-time dynamics [146]. While emerging technologies such as organ-on-a-chip techniques offer more physiologically relevant platforms to assess organoid function, they also come with notable limitations. These include issues with low imaging resolution and reduced data acquisition speed [146]. Furthermore, the complex nature of organoid culture, especially the variation in biomechanical matrices add complications in designing reliable and standardized algorithms to interpret functional outputs [147]. The complexity hampers the ability of current technologies to generate high-throughput functional readouts necessary for disease modeling.

### 5.4. Ethical and accessibility concerns

The use of organoids raises several ethical concerns, particularly regarding the source and use of human-derived tissue and SCs. A primary concern involves the procurement of donor tissues, where issues of informed consent, donor anonymity, and data protection are of great significance. Donors must be fully informed not only about the intended use of their tissues but also about possible future applications such as gene editing, organoid transplantation, and creation of chimeric models [148]. The risks of misuse (commercial, clinical, or experimental) demand that we establish robust ethical oversight mechanisms. We must exercise particular caution and intense ethical scrutiny when dealing with human-animal chimeric organoids, with the gravest consideration reserved for models involving human brain tissues. Some scholars argue that such constructs, particularly if they show advanced cognitive potential, should be afforded greater ethical consideration than other animal models. For instance, it has been proposed that animals showing signs of higher cognitive function after engraftment with human brain organoids should be humanely euthanized at the conclusion of the study [139]. Similarly, the transplantation of human gonadal organoids into animals raises concerns about cross-species reproduction, and donors may withdraw consent if their tissues are used in such experiments. In addition to the above concerns, ethical considerations also arise around patient privacy, cultural values, and respect for religious traditions. The use of tissues obtained from the deceased may conflict with cultural or religious beliefs, further complicating tissue acquisition and public acceptance [130].

## 6. Future directions

Recent advances in organoid models have significantly narrowed the gap between traditional cell line models and *in vivo* systems. These improvements have enhanced basic biological research, clinical applications, and translational studies, bridging the laboratory and clinical settings. As scientific advancement moves toward personalized treatment modalities, organoids offer valuable potential by enabling more accurate drug screening and development. For instance, tumor-derived organoids, when integrated with multi-omics data, can predict drug sensitivity and support personalized treatment strategies [144]. Despite current limitations associated with organoid culturing conditions, several innovative approaches are being developed to improve organoid accuracy. One major limitation is that the lack of vasculature within organoids is addressed by developing both *in vivo* and *in vitro* vascularization approaches. *In-vivo* vascularization techniques include transplanting organoids into animal models to stimulate natural blood vessel formation, while the *in-vitro* vascularization approach combines gene editing with microfluidic platforms to generate vascularized environments [144]. Efforts to enhance cell-cell and cell-matrix interactions are another limitation of organoid culture, which is tackled by optimizing hydrostatic and hydrodynamic forces, as well as introducing spatial and temporal regulations within organoid systems using biosensors, organ-on-chip platforms and other bioengineering techniques [147]. Further advancements in organ-on-chip systems, especially the development of single-organ microarrays or small multi-organ microarrays, offers promising avenues to replicate complex physiological environments. While these advancements may introduce additional complexity in terms of culturing conditions, they bring organoid systems closer to mimicking native tissue functionality and inter-organ interactions [146].

The development and progression of CRC are strongly associated with the intestinal microbiome, but in CRC organoids, mimicking the TME remains a challenge. Generating CRC organoids that incorporate both the dynamic behavior of cancer cells and the complexity of TME, including microbiome interactions and bi-directional crosstalk, will extend functional insights into treatment response and drug-resistance mechanisms [131]. Additionally, establishing PDOs will further contribute to studying the genetic and phenotypic diversity of tumors [131]. This will be significant in refining precision medicine approaches, improving treatment efficacy, and reducing adverse effects by tailoring therapies to individual patient profiles.

As organoid technologies continue to advance, their integration with multi-omics and bioengineering platforms and improved culturing conditions will further close the gap between *in vitro* and *in vivo* models. These advancements will enhance our understanding of disease mechanisms and pave the way toward more predictive and personalized therapeutic strategies.

## Data Availability

The data that support the findings of this study are available upon reasonable request from the authors. This is a review paper. So that, data is not associated with this manuscript

## Fundings

This project is supported by: (1) Grant # RP210153 (to SR) from the Cancer Prevention & Research Institute of Texas (CPRIT). (2) Grant # 5U54MD007592 Pilot Project (to SR) from the National Institute on Minority Health and Health Disparities (NIMHD), a component of the National Institutes of Health (NIH). (3) Grant # OT2OD034190 (to SR) from the NIH Cloud Platform Interoperability Administrative Coordinating Center (NCPI-ACC). (4) Start-up funds (to SR) from University of Texas at El Paso, (5) Grant # RP230446 from the Cancer Prevention & Research Institute of Texas (CPRIT) (Prof. Weiqin Lu), which supports Md Zahirul Islam Khan.
